# Cytotoxicity, antimicrobial and antioxidant activities of mosses obtained from open habitats

**DOI:** 10.1371/journal.pone.0257479

**Published:** 2021-09-20

**Authors:** Grzegorz J. Wolski, Beata Sadowska, Marek Fol, Anna Podsędek, Dominika Kajszczak, Agnieszka Kobylińska

**Affiliations:** 1 Department of Geobotany and Plant Ecology, Faculty of Biology and Environmental Protection, University of Lodz, Łódź, Poland; 2 Department of Immunology and Infectious Biology, Institute of Microbiology, Biotechnology and Immunology, Faculty of Biology and Environmental Protection, University of Lodz, Łódź, Poland; 3 Institute of Molecular and Industrial Biotechnology, Faculty of Biotechnology and Food Sciences, Lodz University of Technology, Łódź, Poland; 4 Department of Plant Ecophysiology, Faculty of Biology and Environmental Protection, University of Lodz, Łódź, Poland; Bangabandhu Sheikh Mujibur Rahman Agricultural University, BANGLADESH

## Abstract

Mosses are mainly the object of ecological and taxonomic research. This group of plants are still underestimated by scientists in other aspects of research. Recent research has shown that these plants contain remarkable and unique substances with high biological activity. Five species of mosses from a large urban ecosystem were identified for present study. In order to determine their biological potential, multifaceted studies were carried out, including: total phenolics content, antioxidant activity, antimicrobial and antifungal study, cytotoxicity evaluation, and scratch assay to assess pro-regenerative effect in the context of their possible use as the ingredients of biologically active cosmetics. Additionally, determination of individual phenolic compounds in selected extracts of the tested mosses was made. Research showed that *Ceratodon purpureus* and *Dryptodon pulvinatus* extracts had the greatest potential as antioxidants and antimicrobial activity. The cytotoxicity assessment indicated that the extracts from *Dryptodon pulvinatus* and *Rhytidiadelphus squarossus* exerted the strongest negative effect on mouse fibroblast line L929 viability at higher concentrations. While, the extract from *Tortulla murali*s best stimulated human foreskin fibroblast line HFF-1 proliferation and wound healing. The research on individual phenolic compounds content in the extracts tested indicated over 20 peaks on UPLC chromatograms. The conducted study has shown that mosses, especially so far unexplored species of open ecosystems, and e.g. epilytic habitats, may be a valuable source of biologically active substances and thus may constitute important medical and cosmetic possibilities.

## Introduction

Mosses are a group of plants widely distributed worldwide but still poorly researched. Currently, it is estimated that there are approximately 22,000–25,000 of these organisms all over the World [1, 2]. Bryophytes are also a very diverse and non-homogeneous group of plants, which in the latest taxonomic studies has been divided into three separate phyla: mosses (Bryophyta Schimp.), liverworts (Marchantiophyta Stotler & Stotl.-Crand.), and hornworts (Anthocerotophyta Stotler & Stotl.-Crand.) [3, 4].

Bryophytes have rarely been the object of human interest. This is mainly due to their low calorific value and poor organoleptic properties, which makes them unusable as a food source [5]. However, bryophytes are used as animal feed in the circumpolar regions [3]. Since these little plants require quite good optical equipment for proper identification, their common recognition, and thus learning about them seem to be also hindered. The above-presented facts have led to a situation where bryophytes are now considered as useless to humans, and even their ecological role is underestimated [3, 5]. However, some scientists appreciate their importance. Bryophytes are most often used in ecological research due to their high bioindicative value relative to the plant communities in which they develop [6–9] or the substrates on which they grow [10–13]. In addition, some species are also considered to be outstanding indicators of the naturalness of plant ecosystems, mainly forest ecosystems [14–16].

Although the interest in the use of bryophytes in medicine goes back only the last few decades [17, 18], these plants have been used as natural medication for centuries (mainly in Asian countries) [19]. In the latest decade, the interest in these plants has been significantly growing due to the discovery that bryophytes, similarly to vascular plants, produce many chemical compounds showing high biological activity [2, 17, 20, 21]. Numerous studies concerning tissues of bryophytes have documented, among others, the presence of hydrocarbons and aromatic compounds (e.g., bibenzyls) along with polycyclic aromatic. Varied groups of organic compounds such as: flavonoids, including flavones (e.g., apigenin, luteolin), flavonols (e.g., kaempferol), isoflavones and other hydroxy flavonoids; terpenoids, including lipophilic mono-, sesqui-, and diterpenoids; glycosides: glycosides of three- and tetraoxygenated coumarins, orobol glycosides; lipids and fatty acids and other volatile constituents have been identified [22–29]. Therefore, well-expressed antibacterial, antifungal, antiviral activities have been demonstrated for a number of bryophytes. Their cytotoxicity with respect to cancer cells, antiplatelet, antithrombin, vasopressin antagonist, cardiotonic, allergic, tumor effecting, insecticidal, molluscicidal, plant growth regulatory and neuroprotective activities, as well as the ability to inhibit a number of biochemically important enzymes have been also confirmed in many studies. Bryophytes exhibit antioxidant and UV photoprotective properties too. Due to the phenolic content, including flavonoids and hydroxycinnamic acids, they can reduce direct and indirect effects of damaging UV radiation penetrating their tissues [1, 5, 17, 20, 21, 26, 27, 29–31, 32–40]. Mosses and their extracts so far have found application, among others, in the treatment of: skin diseases and skin-associated problems; respiratory system diseases; and cardio vascular system diseases. Various types of bryophytes have also antipyretic and diuretic properties, therefore they have been used to treat: hepatitis, fractures, tonsillitis, neurasthenia, viral diseases, and other illnesses [1, 2, 33, 34, 40–45].

Recent studies have demonstrated that many compounds isolated from bryophytes reveal high biological activity [32, 33, 46]. However, although 3,000 bryophytes are reported to have medicinal value, only few have been developed for medical use [29, 34, 37]. Conducted for several decades studies on the use of bryophytes as a potential source of biologically active substances have focused mainly on liverworts, much less often on mosses or hornworts [2]. Reported research usually concentrates on moss species common mostly in the epigeic forest [5, 21, 29, 31, 34–38, 47–51]. The species from other ecological groups, and also from open habitats are often overlooked. Due to the lack to our knowledge of this sort of data the authors have undertaken research aimed at the characterization and determination of the medicinal potential of widespread mosses collected from open habitat areas of large urban ecosystems: *Ceratodon purpureus* (Hedw.) Brid., *Dryptodon pulvinatus* (Hedw.) Brid., *Hypnum cupressiforme* Hedw., *Rhytidiadelphus squarossus* (Hedw.) Warnst., *Tortulla murali*s Hedw. as possible ingredients of biologically active cosmetics / pharmaceutics to support the treatment of skin disorders and wound healing.

## Material and methods

### Plant material

**Fig 1:**
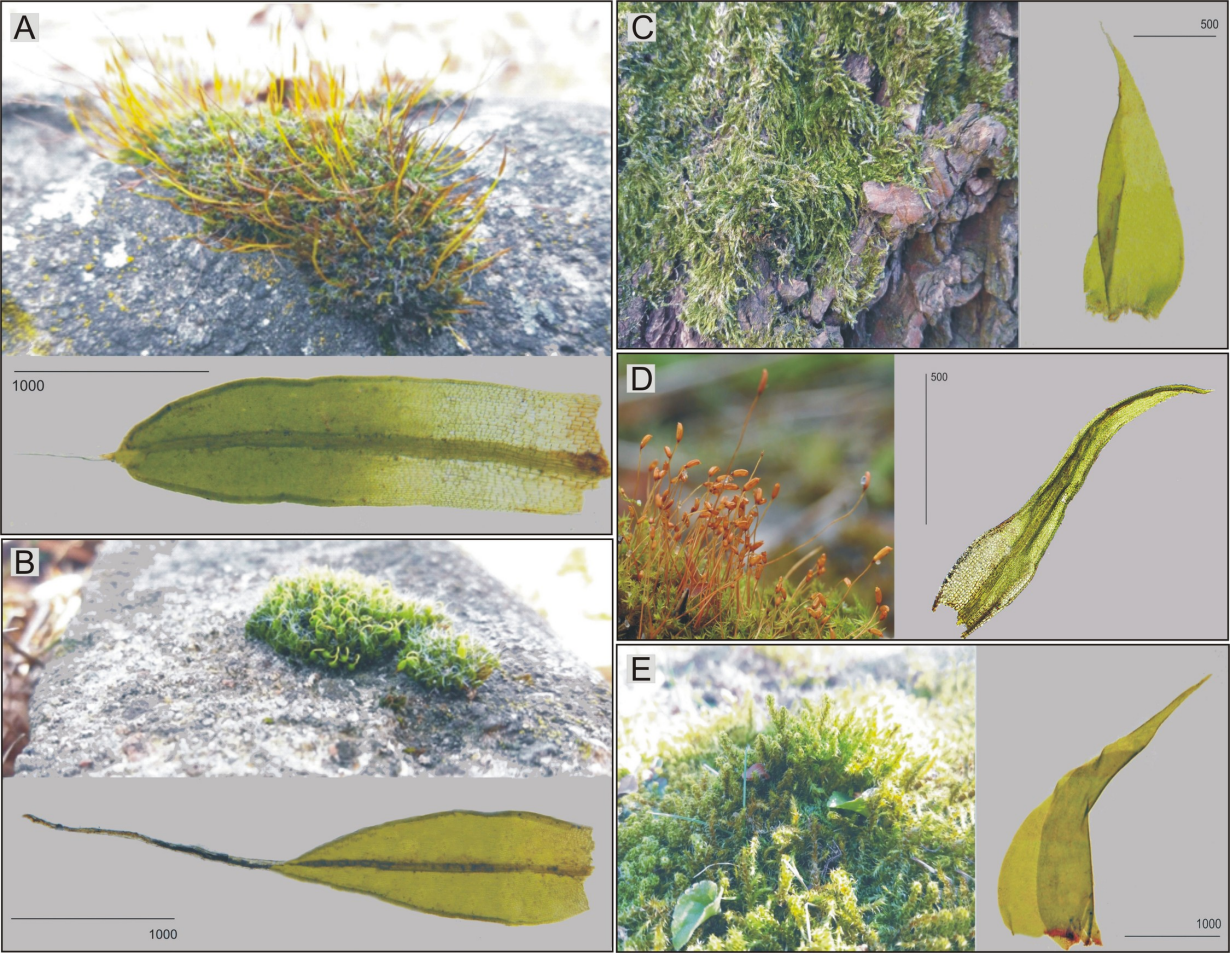
Studied moss species, their stem leaf and habitat. A–*T*. *muralis*, B–*D*. *pulvinatus*, C–*H*. *cupressiforme*, D–*C*. *purpureus*, E–*R*. *squarrosus*; scale in μm (foto. G. J. Wolski, Scientific and Didactic Garden, Faculty of Biology and Environmental Protection, University of Lodz, 08.03.2019).

Plants were collected from the area of the Scientific and Didactic Garden, Faculty of Biology and Environmental Protection, University of Lodz, and its immediate surroundings, in the spring of 2018. The species common for this area, covering all available habitats, were selected for this research: epilithic species (*Tortula muralis* and *Dryptodon pulvinatus*, Fig 1A and 1B), epiphytic species (*Hypnum cupressiforme* from *Salix alba* bark, Fig 1C), and epigeic species (growing on soil *Ceratodon purpureus* and lawn *Rhytidiadelphus squarrosus*, Fig 1D and 1E), and a detailed description of the selected species along with taxonomic features is presented in S1 Appendix.

### Extraction procedure

The weighed fresh plant material was air-dried at 40°C for 24 h, ground were ground in a mortar with a pestle to the powder and extracted with pure ethanol at a solid to liquid ratio 1:10 (w/v) for 30 min, at room temperature on the rocker. The supernatant was decanted and the sediment was boiled 2 min. with pure ethanol. The combined ethanol extracts were centrifuged for 10 min at 4500 × *g*, 4°C (3K30, Sigma). After removal of ethanol at ≤ 40°C with a vacuum rotary evaporator (RV06-ML IKA- WERKE, Germany) the extracts were reconstituted at 1 mg/mL of 5% ethanol.

### Total phenolics content

Total phenolic content was estimated by the Folin-Ciocalteu method using gallic acid as a standard [52]. 500 μL of the extract were combined with 3.65 mL of distilled water, 100 μL of Folin-Ciocalteu’s reagent and 1000 μL of 10% Na_2_CO_3_. The mixture was vortexed thoroughly and, after incubation at room temperature in darkness for 60 min, the absorbance was measured at 765 nm against a ‘blank’ without the sample extract (UV-Vis spectrophotometer U-2001, Hitachi). Quantification was done on the basis of the standard curve of gallic acid (solution 0.25–5 μg/mL). The results were expressed as mg of gallic acid equivalents (GAE) per g of fresh weight (FW). All raw data is added as S1 Raw data.

### 2,2′‐azinobis (3‐ethylbenzthiazoline‐6‐sulphonic acid) radical cation decolouration assay

A modified method of Re et al. [53, 54] was used. Briefly, a fresh solution of ABTS^·+^ was prepared by dissolving 19.5 mg 2,2′‐azinobis(3‐ethylbenzthiazoline‐6‐sulphonic acid) (ABTS; Sigma, Deisenhofen, Germany) and 3.3 mg potassium persulphate (dipotassium peroxodisulphate; Sigma) in 7 mL of 0.1 mol/L phosphate buffer, pH 7.4. This solution was stored in the dark for 12 h for completion of the reaction. A 50 μL of ethanolic extract of bryophytes was added to 2 ml ABTS^·+^ solution in 0.1 mol/L phosphate buffer, pH 7.4, diluted (usually approximately 1:80) to give an absorbance about 0.9 and read at 734 nm. The extent of ABTS^·+^ bleaching (decrease in absorbance, corrected for a small decrease in absorbance of ABTS^·+^ solution alone) is proportional to the activity of anti-oxidants in a given sample. Calculations were made on the basis of standard curves obtained for a Trolox solution. Antioxidant capacity of plant extracts were expressed as Trolox equivalent [μM]. All raw data is added as S1 Raw data.

### Ferric ion reducing anti-oxidant power assay

For the ferric ion reducing anti-oxidant power (FRAP) assay, a modification of the method of Benzie and Strain was used [55, 56]. Working solution was prepared immediately before measurements by mixing 10 volumes of acetate buffer, pH 3.6, with 1 volume of 10 mM/L 2,4,6–tris-2-pyridyl-s-triazine (TPTZ; Sigma) and 1 volume of 20 mM/L FeCl_3_ (Sigma). A 50 μL of extracts were mixed with 2 mL of the working solution and incubated at 37°C. After 5 min, the absorbance of the samples was read at 593 nm in a spectrophotometer against a reagent blank. The increase in absorbance is proportional to the activity of antioxidants in the sample. Calculations were made on the basis of standard curves obtained for FeSO_4_ solution. The ferric reducing ability of extracts measured as antioxidant power were expressed as FeSO_4_ equivalent [μM]. All raw data is added as S1 Raw data.

### Microorganisms, culture conditions and suspensions for antimicrobial study

The reference strains of Gram-positive bacteria: *Staphylococcus aureus* ATCC 29213 (MSSA, methicillin-susceptible *S*. *aureus*), *Staphylococcus aureus* ATCC 43300 (MRSA, methicillin-resistant *S*. *aureus*), *Staphylococcus epidermidis* ATCC 12228, *Enterococcus faecalis* ATCC 29212, Gram-negative bacteria: *Escherichia coli* ATCC 25922, *Pseudomonas aeruginosa* ATCC 25619, and fungi: *Candida albicans* ATCC 10231, *Candida glabrata* ATCC 90030 were used to assess biostatic/biocidal activity of the extracts from selected mosses. The group of microorganisms tested (bacteria and fungi) contain standard reference strains used for screening method of antimicrobial activity testing. Microorganisms were grown for 24 h at 37°C in tryptic-soy agar (TSA; BTL, Poland) or Sabouraud dextrose agar (SDA; BTL, Poland) according to nutritional requirements. Microbial suspensions at a density of about 5 **×** 10^5^ CFU/mL were prepared in Mueller-Hinton broth (MHB; BTL, Poland) or in RPMI-1640 medium with L-glutamine (Sigma-Aldrich, Merck, Germany) containing 2% glucose (RPMI/Glu) for bacteria or fungi, respectively.

### Minimum inhibitory and bactericidal/fungicidal concentration (MIC, MBC/MFC)

Broth microdilution method was used according to the EUCAST guidelines [EUCAST] to determine MIC of moss extracts. Tested extracts were diluted in MHB or RPMI/Glu (for bacteria and fungi, respectively) to the final concentration range of 0.015–1.0 mg/mL in 96-well culture plate (Falcon, USA) in volume of 50 μL/well. Then microbial suspensions (5 × 10^5^ CFU/mL, 50 μL) were added and the plates were incubated at 37°C for 24 h (bacteria) or 48 h (fungi). Since primary *D*. *pulvinatus* and *R*. *squarrosus* extracts were prepared in 100% Et-OH at a concentration of 40 mg/mL and the highest concentration of the solvent in tested wells with 1 mg/mL of these two extracts reached 2.5%, two types of microbial growth positive control were simultaneously prepared: microbial suspensions in medium alone (K + 1) and microbial suspensions in medium containing 2.5% Et-OH (K + 2). MIC was defined as the lowest concentration of the extract inhibiting visible bacterial/fungal growth during of co-incubation time compared to the appropriate positive control. MBC/MFC of the extracts tested referred to the lowest concentration that killed 99.9% of microbial inoculum added to the wells, thus there was no bacteria or yeast growth after subculturing 10 μL from the wells marked as MIC and these containing higher concentrations on TSA/SDA (incubation 24–48 h at 37°C). Experiments were carried out in duplicate for each extract.

### Cytotoxicity evaluation of the extracts for mouse fibroblasts line L929 by MTT method

Mouse fibroblasts L929 (ATTC, CCL-1) were cultured as monolayers in 25 cm^2^ tissue culture flasks (BD Falcon) in RPMI 1640 medium (Sigma/Merck, Germany) supplemented with 10% fetal bovine serum (Biological Industries) and 100 U/mL penicillin, 100 μg/mL streptomycin, 0,292 mg/mL L-glutamine (Gibco/Intvitrogen). Every three days the monolayer was treated with trypsin/ethylene diamine tetraacetic acid (BioWest) and sub-cultured. Cells were maintained at 37°C under a humidified atmosphere containing 5% CO_2_. Cytotoxicity was assessed using the colorimetric MTT assay [57]. Briefly, confluent monolayer was trypsinized, the suspension of 1 × 10^5^ cells/mL prepared, 100 μL per well of 96-well tissue culture plate (Greiner bio-one) seeded, and incubated at 37°C in a humidified 5% CO_2_ atmosphere. For background absorption, some wells were remained cell-free (blank control). After 24h the growth medium was discarded, and 100 μL of extract diluted in RPMI 1640 medium (supplemented as earlier) was added at the following concentrations: 1000, 500, 250, 125, 62.5, 31.2, 15.6, and 7.8 μg/mL (n = 3). In each case the concentration of extracts’ solvent (Et-OH) did not exceed 2,5% and did not disturb fibroblast growth. Three wells were remained untreated as negative control. Following 24 h incubation under the conditions as earlier, the treatment medium were removed and 50 μL/well of MTT ((3-(4,5-dimethylthiazol-2-yl)-2,5-diphenyltetrazolium bromide; Sigma/Merck) in phosphate-buffered saline (1 mg/mL) after filtration through a 0.2 μm syringe filter was added and incubated for 2 h as above. The MTT solution was removed and replaced with 150 μL/well of DMSO (dimethyl sulfoxide; Sigma/Merck) to dissolve the blue formazan product formed by living cells. Additionally, 25 μL/well of Sörensen’s glycine buffer (glycine 0.1 M, NaCl 0.1 M, pH:10.5 with 0.1 NaOH) was added. The absorbance (A) was detected at 570 nm with the use of the microplate reader Multiskan EX (Thermo Labsystem-Fischer, USA). The viability of cells cultured in full RPMI 1640 alone (negative control) was considered as 100%, and the percentage of cell viability was calculated using the formula below:

Survival rate (%) = [(As–Ab)/(Ac–Ab)] × 100
As–absorbance of test sample (the cells exposed to the extract)
Ac–absorbance of negative control (the cells in medium alone)
Ab–absorbance of blank control
All raw data is added as S2 Raw data.

### Determination of individual phenolic compounds using UPLC/Q-TOF-MS analysis

Caffeic acid, (+)-catechin, apigenin, naringin, formic acid, methanol and acetonitrile were obtained from Sigma Aldrich (Steinheim, Germany). Chlorogenic acid, quercetin 3-glucoside, quercetin 3-rutinoside, kaempferol 3-glucoside and *p*-coumaric acid were obtained from Extrasynthese (Lyon, France). Procyanidin B1 and 3,5-dicaffeoylquinic acid was purchased from PhytoLab (Vestenbergsgreuth, Germany).

Phenolic compounds were identified using the Acquity ultra-performance liquid chromatography (UPLC) system coupled with a quadruple-time of flight mass spectrometry (Q-TOF-MS) instrument (Waters Corp., Milford, MA, USA) equipped with an electrospray ionization (ESI) source. Separations of individual phenolics were carried out using a Acquity UPLC^R^ HSS T3 C18 column (150 × 2.1 mm, 1.8 μm; Corp., Milford, MA, USA) at 30°C, as previously described [58]. The mobile phase was a mixture of 0.1% formic acid (A) and acetonitrile (B). The gradient program was as follows: initial conditions 99% (A), 12 min 65% (A), 12.5 min 0% (A), 13.5 min 99% (A). The flow rate was 0.45 mL/min and the injection volume was 5 μL. The mass spectrometer was operating in the negative mode for a mass range of 150–1500 Da, fixed source temperature at 100°C, desolvation temperature 250°C, desolvation gas flow of 600 L/h, cone voltage of 45 V, capillary voltage of 2.0 kV, collision energy 50 V. Leucine enkephalin was used as a lock mass. The instrument was controlled by Mass-Lynx^TM^ V 4.1 software. Photodiode detector spectra were measured over the wavelength range 200–600 nm. Compounds were identified using their UV-Vis characteristic, MS and MS^2^ properties using data gathered in house and from literature. Calibration curves were run for the external standards: (+)-catechin, procyanidin B1, caffeic acid, chlorogenic acid, 3,5-dicaffeoylquinic acid, *p*-coumaric acid, quercetin 3-glucoside, quercetin 3-rutinoside, kaempferol 3-glucoside, apigenin, and naringin.

The crude extracts prior to injection into UPLC-MS system were extracted twice with chloroform to remove lipophilic compounds. The organic phase was discarded and the aqueous phase was concentrated to 3 mL, and applied to a C18 solid phase extraction cartridge (360 mg capacity, Waters Corp., Milford, MA) to separate phenolic compounds fraction. Interfering water-soluble components were removed with water (10 mL) while absorbed phenolics were recovered with methanol (10 mL). The solvent allowed to evaporate completely under reduced pressure and solid residue was dissolved in 2 mL of methanol.

### Scratch assay

Human foreskin fibroblasts (HFF-1 line; LGC Standards, UK) were culture in 24-well plates (Nunc, Denmark) with previously marked reference lines on wells’ bottom (2.5 **×** 10^5^ cells/well) in Dulbecco’s Modified Eagle Medium (DMEM) supplemented with 15% fetal bovine serum (FBS; Biological Industries, USA) and penicillin-streptomycin solution (P/S; Biowest, France) at 37°C, 5% CO_2_. The 24 h-old cell monolayers were gently scratched across the reference lines, forming the wounds. The medium containing detached cells was removed, the cells were washed twice using culture medium and finally fresh culture medium alone (control cells) or the medium with moss extracts (15 μg/mL) were added. The cells were grown for the next 24 h. The images of the wounds immediately after scratching (T0) and 24 h later (T24) were done using Motic Microscope model AE-2000T with inverted field of view and integrated camera (Conbest Sp. Z o.o., Poland). The changes of total wound areas were analyzed using ImageJ software. The percentage of wound closure was calculated according to the following formula: [(wound area T0—wound area T24) / wound area T0] × 100. Experiment was carried out twice.

## Results

### Total phenolic content

**Fig 2:**
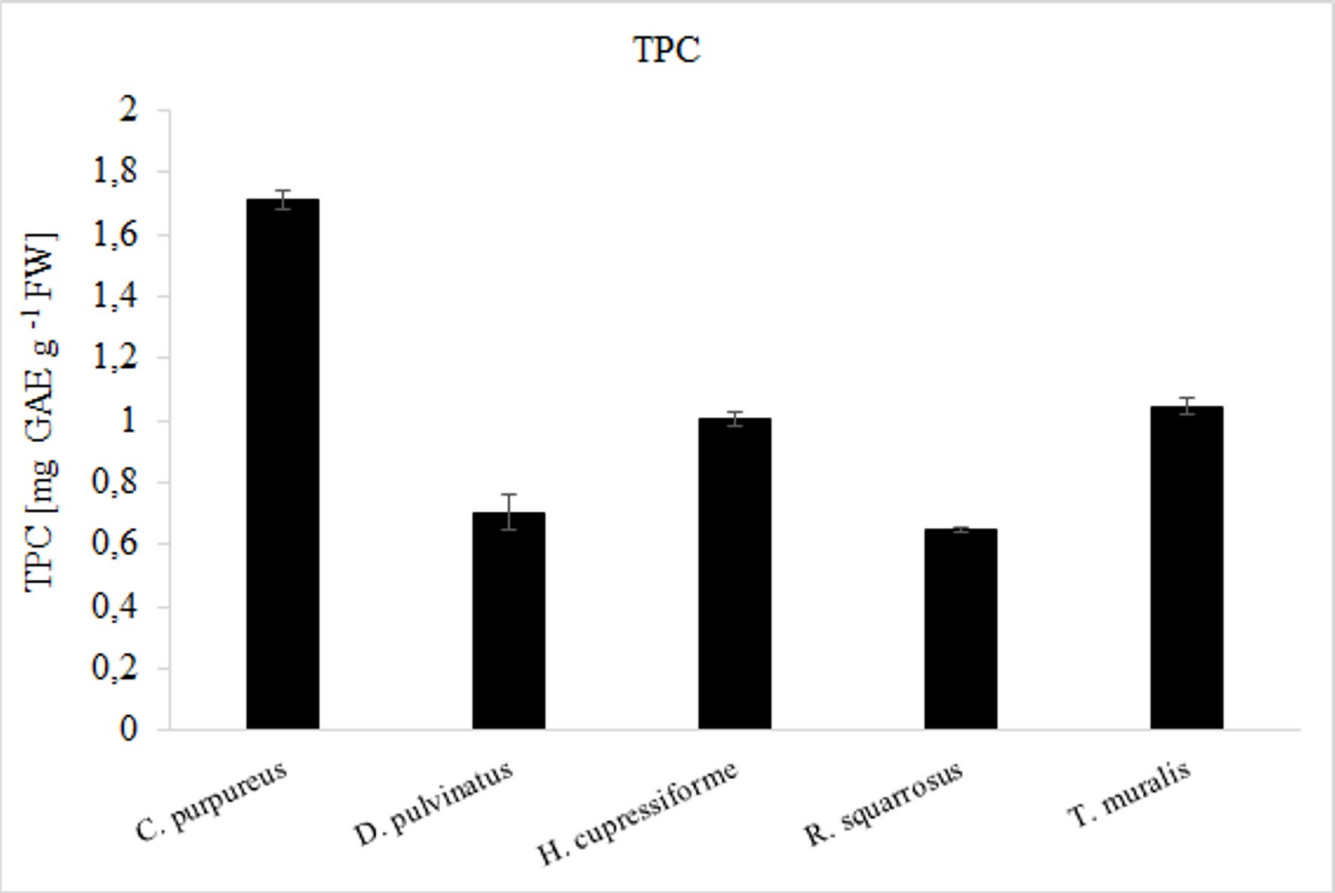
The total phenolic content of selected mosses expressed as mg gallic acid equivalent per g of fresh sample. The results are expressed as mean values of 3 independent experiments ± SD.

The results of the colorimetric analysis of total phenolic content (TPC) based on the absorbance values of the extract solutions reacted with Folin Ciocalteu’s reagent, expressed as mg gallic acid equivalent (GAE) per g FW of sample, are given in Fig 2. The highest values– 1.71 ± 0.03 was noted for *C*. *purpureus*. Significantly lower (by about 40%)– 1,04 ± 0.03 and 1.00 ± 0.02 mg GAE/g FW of sample–were determined for *T*. *muralis* and *H*. *cupressiforme*, respectively. The next species *D*. *pulvinatus* and *R*. *squarossus* had comparable concentration of phenolics, approximately by 60% lower than that obtained for *C*. *purpureus*.

### Antioxidant capacity

**Fig 3:**
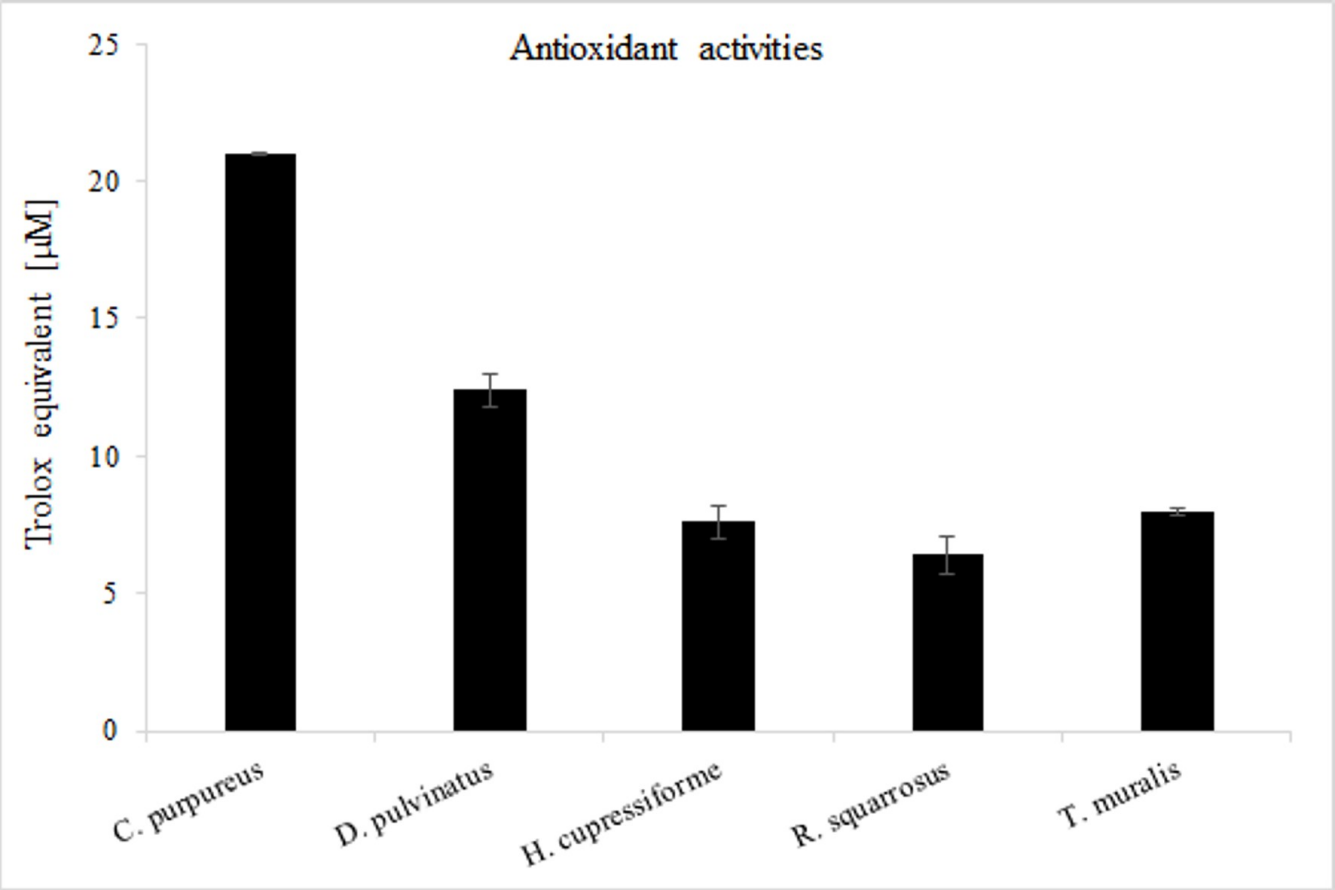
The ABTS cation scavenging activity of selected mosses extracts calculated as Trolox equivalent [μM]. The results are expressed as mean values of 3 independent experiments ± SD.

**Fig 4:**
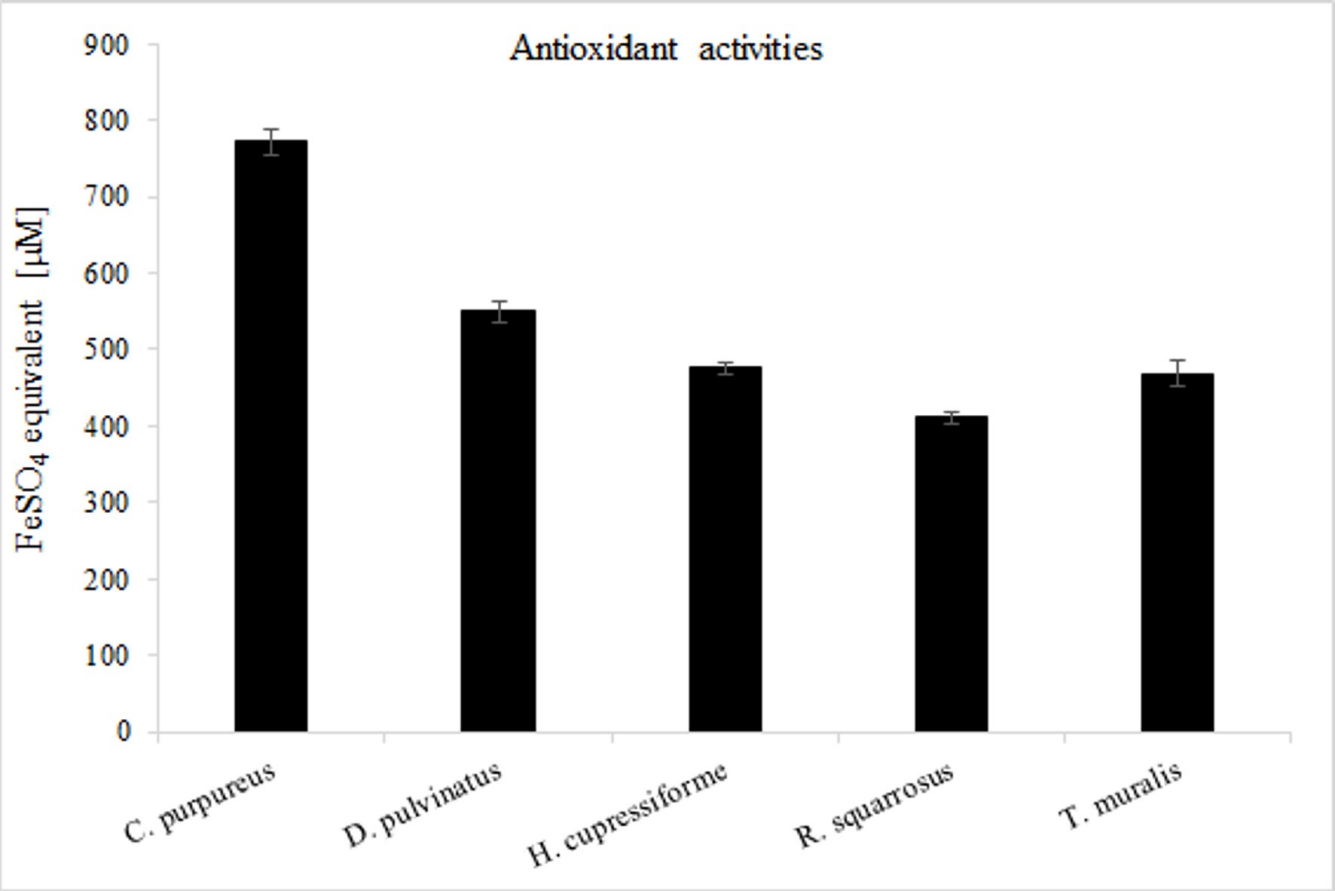
Reducing antioxidant power of mosses extracts calculated as FeSO_4_ equivalent [μM]. The results are expressed as mean values of 3 independent experiments ± SD.

Five plant species used in our experiments were screened for their potential as antioxidants. Due to the fact that different antioxidants had varying reactivity and substrate specificity we applied two various methods: the ferric reducing antioxidant power (FRAP) and the 2,2′‐azinobis‐3‐ethylbenzotiazoline‐6‐sulfonic acid (ABTS^·+^) assay for the measurement of the total antioxidant capacity. Our results indicates that both radical scavenging activity and reducing properties in analyzed extracts decreased in the following order: *C*. *purpureus* > *D*. *pulvinatus* > *T*. *muralis* > *H*. *cupressiforme* > *R*. *squarrosus*. Trolox equivalent for *C*. *purpureus* was 40% higher than for *D*. *pulvinatus* and over 60 and 70% higher in comparison to *T*. *muralis*, *H*. *cupressiforme* and *R*. *squarrosus*, respectively (Fig 3). Similar tendency was observed using FRAP method. The ferric ion reducing anti-oxidant power determined for *C*. *purpureus* compared to *D*. *pulvinatus*, *T*. *muralis*, *H*. *cupressiforme and R*. *squarrosus* was better about 30%, 40% and 50%, respectively (Fig 4).

### Antimicrobial activity

The antimicrobial activity of moss extracts against selected bacterial and fungal strains are presented in detail in Table 1 as minimum inhibitory concentration (MIC) and minimum bactericidal/fungicidal concentration (MBC/MFC). The moss extracts showed no biostatic and biocidal activity in whole concentration range tested (up to 1 mg/mL). The only exception was the *D*. *pulvinatus* origin extract, which inhibited the growth of *C*. *glabrata* at a concentration of 0.5 mg/mL. However, the lack of similar effect against *C*. *albicans* starts a discussion about antifungal properties of this extract, which need further investigations.

**Table 1:** The antimicrobial activity of mosses extracts.

Microorganism	MIC [mg/mL]				
	MBC/MFC [mg/mL]				
	*T*. *muralis*	*H*. *cupressiforme*	*C*. *purpureus*	*D*. *pulvinatus*	*R*. *squarrosus*
**Bacteria**					
*Staphylococcus aureus* ATCC 29213	> 1	> 1	> 1	> 1	> 1
	> 1	> 1	> 1	> 1	> 1
*Staphylococcus aureus* ATCC 43300	> 1	> 1	> 1	> 1	> 1
	> 1	> 1	> 1	> 1	> 1
*Staphylococcus epidermidis* ATCC 12228	> 1	> 1	> 1	> 1	> 1
	> 1	> 1	> 1	> 1	> 1
*Enterococcus faecalis* ATCC 29212	> 1	> 1	> 1	> 1	> 1
	> 1	> 1	> 1	> 1	> 1
*Escherichia coli* ATCC 25922	> 1	> 1	> 1	> 1	> 1
	> 1	> 1	> 1	> 1	> 1
*Pseudomonas aeruginosa* ATCC 25619	> 1	> 1	> 1	> 1	> 1
	> 1	> 1	> 1	> 1	> 1
**Fungi**					
*Candida albicans* ATCC 10231	> 1	> 1	> 1	> 1	> 1
	> 1	> 1	> 1	> 1	> 1
*Candida glabrata* ATCC 90030	> 1	> 1	> 1	0.5	> 1
	> 1	> 1	> 1	> 1	> 1

MIC–minimum inhibitory concentration measured by broth microdilution method; MBC/MFC—minimum bactericidal/fungicidal concentration determined based on microbial culture on solid media.

### Cytotoxicity evaluation

**Fig 5:**
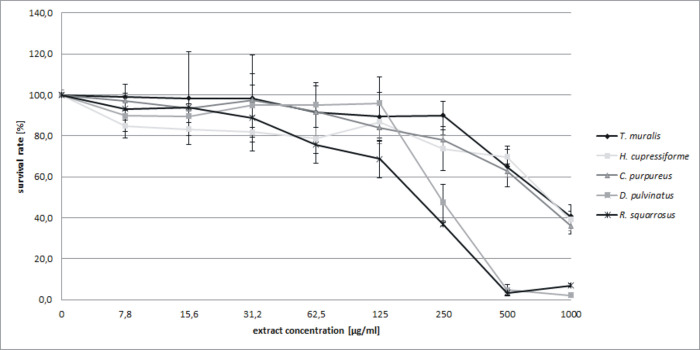
Dose dependent effects of ethanolic moss extracts on L929 cells after 24 h of exposure. Cell viability was quantified using MTT colorimetric method. The results are expressed as mean ± SD. Statistical significance (p < 0.05) was calculated with Kruskal-Wallis test.

**Fig 6:**
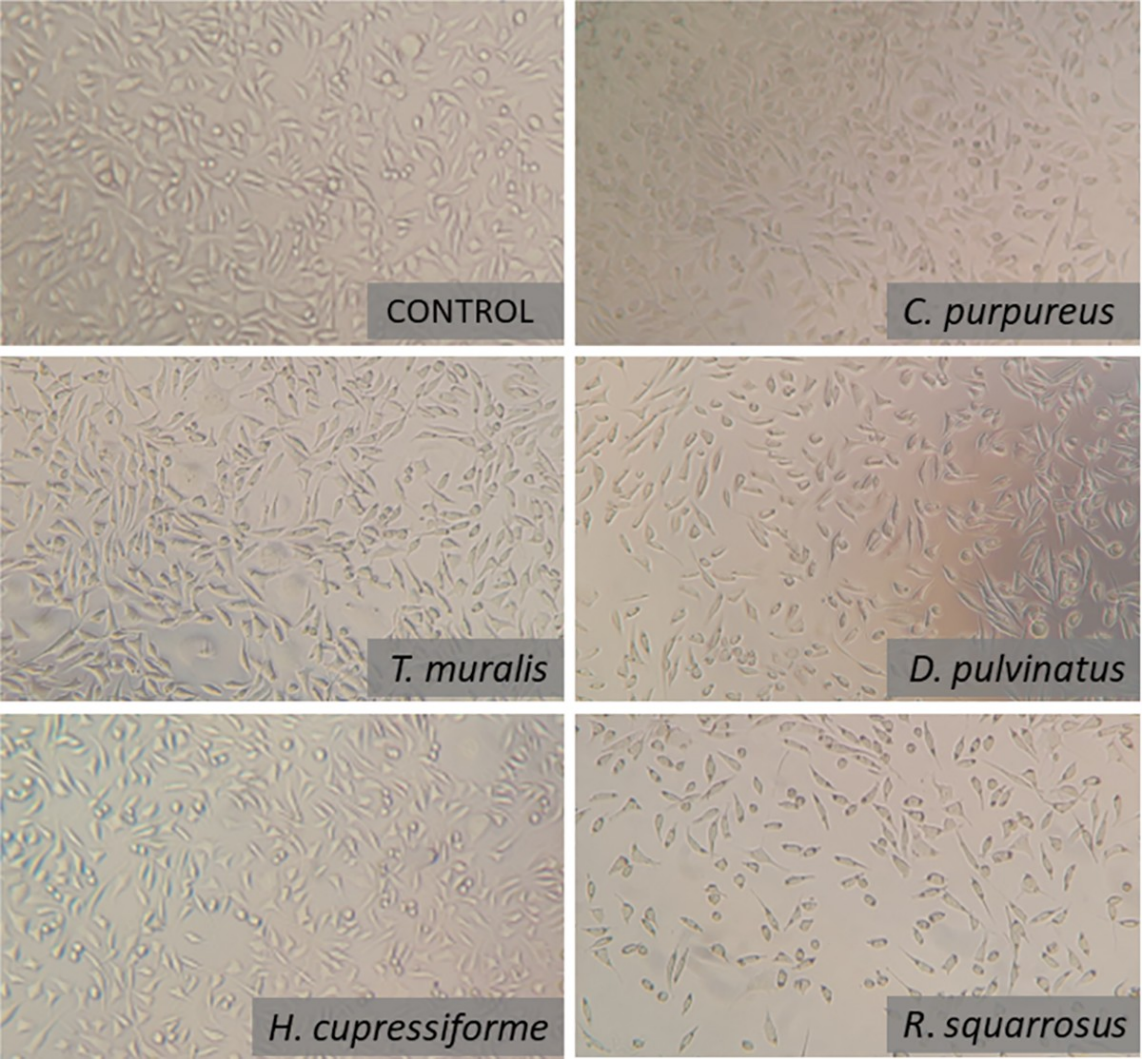
The effect of moss extracts used at the concentration 250 μg/mL on L929 cell monolayer. The images were taken by inverted light microscopy after 24 h exposure of the cells to the extracts.

The cytotoxicity effect of five moss extracts was analyzed after 24 h exposure of L929 cells to tested preparations using MTT assay. The results showed that all tested extracts did not affect the viability of L929 cells at the concentration up to 125 μg/mL, and up to 500 μg/mL for *T*. *muralis* and *H*. *cupressiforme* extracts. The most unfavorable effect was noted for *R*. *squarrosus* extract with cell viability at the level of 68.6 ± 9.2% when used at 125 μg/mL. Culturing of L929 at higher concentrations of both *R*. *squarrosus* and *D*. *pulvinatus* extracts resulted in a sharp decrease in the cells viability (Fig 5) and caused reduced confluence of the monolayer (Fig 6). The extracts from *T*. *muralis*, *H*. *cupressiforme* and *C*. *purpureus* used at the highest concentration tested (1000 μg/mL) were also cytotoxic, however nearly 40% of cells were still viable, whereas the cell viability did not exceed 10% in the case of *D*. *pulvinatus* and *R*. *squarrosus* used at the same concentration.

## Identification and content of phenolic compounds with UPLC–PDA-Q/TOF-MS method

Due to the strongest effect of *D*. *pulvinatus* and *R*. *squarrosus* extracts on eukaryotic cells, including also potential antifungal activity, a detailed chemical composition in relation to individual phenolic compounds was tested for these two extracts.

**Fig 7:**
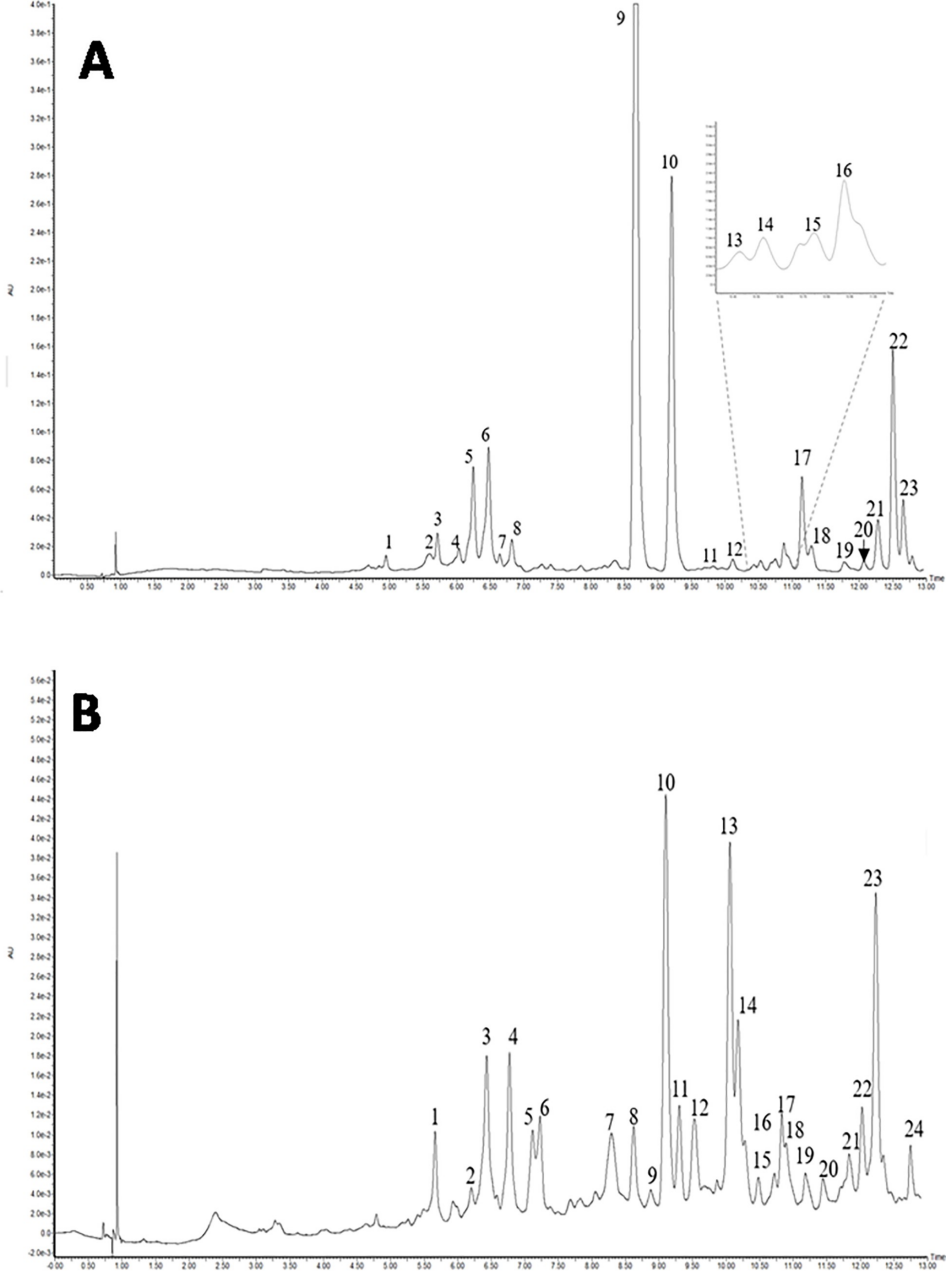
UPLC chromatograms of *D*. *pulvinatus* (A) and *R*. *squarrosus* (B) at 280 nm.

The results of qualitative analysis of extracts determined by UPLC/MS method are presented in Fig 7 and Tables 2 and 3.

**Table 2:** Profile of phenolic compounds in *D*. *pulvinatus* determined by UPLC-ESI-MS/MS analysis.

Peak	R_t_	λ_max_	[M−H]^−^	MS/MS	Compound
	(min)	(nm)		(m/z)	
			(m/z)		
1	4.95	243, 283	519	164,192,221,136,182	Unidentified
2	5.59	243, 288	371	119,124	Unidentified
3	5.72	243, 292	499	174,145,129,187	Unidentified
4	6.03	243, 284	499	174,145,129	Unidentified
5	6.25	243, 340	345	153,137,181,137	Unidentified
6	6.48	243, 284	329	123,153	Unidentified
7	6.64	243, 288	355	148,165,132,227,121,152	Unidentified
8	6.82	243, 314	507	153,181,182,137	Unidentified
9	8.67	243, 284	345	153,181,139,111	Unidentified
10	9.20	280, 335	337	174,175,163,129	Coumaroylquinic acid^a^
11	9.69	243, 345	447	284,133,297,175	Kaempferol 3-galactoside^a^
12	10.11	243, 283	539	298,299,327,297	Unidentified
13	10.43	243, 283	449	135,151,107	Eriodyctiol hexoside^a^
14	10.53	243, 326	431	268,117,283,161,163	Apigenin hexoside^a^
15	10.75	243, 345	609	271,300,255,243,151	Quercetin 3-rutinosdie
16	10.88	243, 347	593	285	Kaempferol 3-rutinoside^a^
17	11.15	243, 347	447	284,227,133,256,199	Kaempferol 3-glucoside
18	11.28	243, 310	505	138,165,214,137	Unidentified
19	11.78	243, 283	449	135,151	Eriodictyol hexoside^a^
20	12.07	243, 280	577	289,151,269,327,343	Procyanidin dimer^a^
21	12.29	279, 338	447	133,285,151,199,179	Kaempferol derivative I^a^
22	12.50	266, 336	447	133,285,151,175,199	Kaempferol derivative II ^a^
23	12.66	267, 324	431	269,239,211,151	Sinapoyl hexoside^a^

^a^–Tentatively identified [58, 59].

**Table 3:** Profile of phenolic compounds in *R*. *squarrosus* determined by UPLC-ESI-MS/MS analysis.

Peak	R_t_	λ_max_	[M−H]^−^	MS/MS	Compound
	(min)	(nm)	(m/z)	(m/z)	
1	5.66	243, 286	371	119	Unidentified
2	6.20	243, 281	289	109,123,108,159,155	(+)-Catechin
3	6.43	243, 281	329	153,119,162	Unidentified
4	6.77	325	353	153,181,137	Chlorogenic acid
5	7.11	282, 322	179	134,108	Caffeic acid
6	7.23	243	447	145,173,211	Unidentified
7	8.28	243	431	138,153,189,205,163,122	Unidentified
8	8.62	243, 284	345	153,137,181	Unidentified
9	8.89	243	387	182,139,153	Unidentified
10	9.10	243, 308	439	131,101,113,175,147	Unidentified
11	9.30	243, 276	433	138,153	Unidentified
12	9.53	243, 283	539	142,172,114,185,317,203	Unidentified
13	10.05	243, 285	167		Unidentified
14	10.18	243, 283	297	101,159,113	Unidentified
15	10.47	243	433	283,117,161	Unidentified
16	10.72	243, 345	609	271,255,300,243,151	Quercetin 3-rutinoside
17	10.83	243, 347	593	285,199,151	Kaempferol 3-rutinoside^a^
18	10.89	243, 338	431	117,281,161,253	Apigenin hexoside^a^
19	11.18	243	505	138,165,211,269	Unidentified
20	11.44	243, 280	431	245,187,202,159,145	Unidentified
21	11.77	283, 320	515	191,135,251,115	3,5-Dicaffeoylquinic acid
22	12.02	243, 281	521	281,254,159,299	Unidentified
23	12.23	243, 279	415	145,241	Unidentified
24	12.74	243, 345	607	284,299,256	Kaempferol derivative II^a^

^a^–Tentatively identified [58, 59].

Chromatogram of *D*. *pulvinatus* showed twenty three peaks, five of which were identified based on retention times (R_t_), Uv-Vis spectra, deprotonated molecules ([M-H]^-^), diagnostic fragments (MS/MS), comparison with the standard reference compounds, and the tentative identification of 3 compounds was based on the literature data. In contrast, chromatogram of *R*. *squarrosus* showed twenty four peaks, but only half of them could be identified, while the tentative identification of as many as ten compounds was based on literature data [58, 59]. Both extracts showed the presence of different groups of phenolic compounds, such as hydroxycinnamic acids, flavanols, flavonols, flavones, and flavanones. The sum of the identified phenolic components in *D*. *pulvinatus* and *R*. *squarrosus* was 25.49 and 6.42 mg/100 g FW of plant material, respectively (Table 4). Quantitatively in the group of identified phenolic compounds, kaempferol derivatives and sinapoyl hexoside were the dominant compounds in *D*. *pulvinatus* while apigenin hexoside and chlorogenic acid in *R*. *squarrosus*.

**Table 4:** Contents of identified phenolic compounds in the extracts of *D*. *pulvinatus* and *R*. *squarrosus*.

Compound	*D*. *pulvinatus*	*R*. *squarrosus*
	Content	
	(mg/100 g of FW)	
Caffeic acid	Not detected	0.30 ± 0.00
Coumaroylquinic acid^a^	0.32 ± 0.00	Not detected
Chlorogenic acid	Not detected	1.26 ± 0.02
3,5-Dicaffeoylquinic acid	Not detected	0.12 ± 0.00
Sinapoyl hexoside^b^	4.42 ± 0.10	Not detected
Kaempferol 3-galactoside^c^	0.22 ± 0.00	Not detected
Kaempferol 3-rutinoside^c^	1.70 ± 0.02	0.69 ± 0.00
Kaempferol 3-glucoside	4.99 ± 0.05	Not detected
Kaempferol derivative I^c^	1.73 ± 0.02	Not detected
Kaempferol derivative II^c^	6.11 ± 0.09	Not detected
Kaempferol derivative III^c^	Not detected	0.28 ± 0.01
Quercetin 3-rutinoside	0.98 ± 0.03	0.70 ± 0.02
Eriodyctiol hexoside I^d^	0.22 ± 0.00	Not detected
Eriodyctiol hexoside II^d^	0.47 ± 0.04	Not detected
Apigenin hexoside^e^	1.93 ± 0.03	2.14 ± 0.17
(+)-Catechin	Not detected	0.93 ± 0.03
Procyanidin dimer^f^	2.40 ± 0.09	Not detected
**TOTAL**	**25.49 ± 0.47**	**6.42 ± 0.27**

Results are expressed as a mean ± standard deviation (n = 3).

^a^–quantified as chlorogenic acid equivalents

^b^–quantified as *p*-coumaric acid equivalents

^c^–quantified as kaempferol 3-glucoside equivalents

^d^–quantified as naringin equivalents

^e^–quantified as apigenin equivalents

^f^–quantified as procyanidin B1 equivalents.

### Pro-regenerative activity of moss extracts

To test pro-regenerative activity of moss extracts *in vitro* scratch assay on human fibroblasts HFF-1 line was used. The moss extracts were applied at very low concentration (15 μg/mL) to avoid any cytotoxic activity.

**Fig 8:**
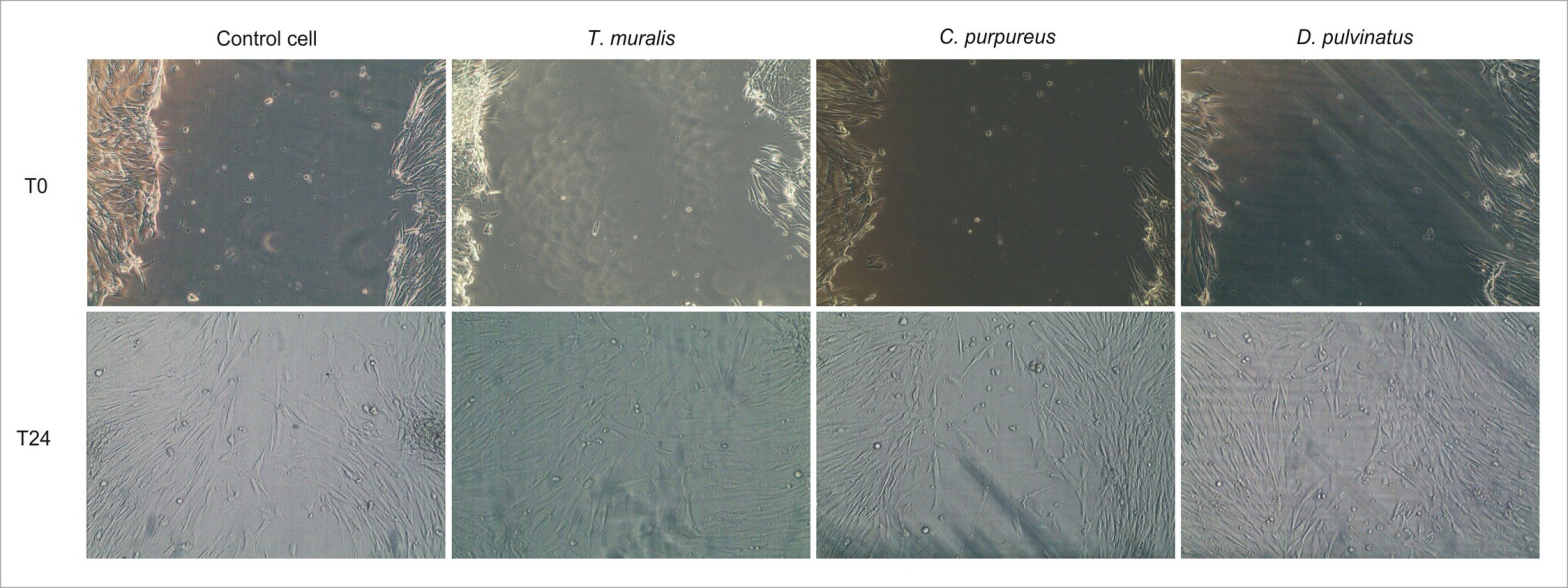
The effect of moss extracts on human foreskin fibroblasts (HFF-1) migration tested *in vitro* by scratch assay. Representative images of the scars taken just after scratching (T0) and 24 h later (T24).

Two groups of moss extracts were used for the study, one group (*T*. *muralis*, *C*. *purpureus*) of preparations with low cytotoxicity (demonstrated only at a concentration above 500 μg/mL) and for comparison, the extract with the highest cytotoxic potential appearing already at a concentration above 125 μg/mL (*D*. *pulvinatus*). The results obtained for three selected extracts representing different models of phytochemical composition and biological activity are presented in Table 5 and Fig 8.

**Table 5:** The pro-regenerative potential of the moss extracts on human foreskin fibroblasts (HFF-1 line) tested *in vitro* by scratch assay.

**Preparation**	**Average wound area at T0 [pixels** ^**2**^ **]**	**Average wound area at T24 [pixels** ^**2**^ **]**	**Average wound closure**
			**[% ± S.D.]**
*T*. *muralis*	2484847	514316	79.3 ± 14.5
*C*. *purpureus*	2461775	919097	62.7 ± 0.5
*D*. *pulvinatus*	2594336	647935	75.0 ± 0.6
Control cells	2320792	793976	66.2 ± 8.6

T0, T24 –time [h] after wound scratch

It was demonstrated, that the extracts with middle and low total phenolic content (from *T*. *muralis* and *D*. *pulvinatus*, respectively) stimulated fibroblasts proliferation and wound healing. The increase in wound closure achieved of 13.1% and 8.8%, respectively, in comparison to control cells. However, pro-regenerative activity cannot be attributed general to moss extracts, since average wound closure was 3.5% lower in the presence of the extract from *C*. *purpureus*, which is characterized, what’s interesting, by the highest total phenolic content.

## Discussion

In the search for new biologically active compounds, mosses can be considered as a largely neglected material, since there have not been many studies dedicated to the composition of secondary metabolites and their biological activity in mosses in comparison to, for example, similar studies of higher plants. Although over 400 years ago, the Chinese used some *Fissidens* and *Polytrichum* species as diuretics and hair growth stimulation tonics. Traditional cultures in India and North America used *Bryum*, *Mnium*, *Philonotis* spp. and *Polytrichum juniperinum* for healing burns, bruises and wounds. *Marchantia polymorpha* was used as a diuretic in France. In many of these cases, a scientific basis has been recently identified to justify use of these plants. Several liverwort and moss extracts have antibacterial, antifungal and antiviral activity [17].

In the recent years bryophytes as a group of plants containing a broad range of biologically active substances, such as phenolics, terpenoids, fatty acids, have been widely tested for pro-health activity. Among beneficial effects of bryophytes *-*origin products (mainly the extracts) anti-oxidative, anti-inflammatory, anti-platelet, anti-leukemic, also antibacterial and antifungal activity are mentioned [2, 36, 37, 60, 61].

In modern cosmetology or phytomedicine the most popular are herbs with the high concentration of phenolics, which is usually reflected in their good antioxidant activity [62, 63]. Antioxidants were also identified in bryophytes tissues, however, so far an attention was focused mainly on liverworts [34, 50], or on epigeic forest mosses species. Mosses harvested from open habitats areas of large urban ecosystem have been omitted, although potentially they can be a rich source of antioxidants [31, 47–49, 51]. Thus in our research five species of widespread mosses harvested from open habitats areas of large urban ecosystem were screened for their medical potential.

Analyses of TPC indicated that phenolics concentration decreased in the following order: *C*. *purpureus* > *T*. *muralis* > *H*. *cupressiforme* > *D*. *pulvinatus* > *R*. *squarrosus*. Generally it is accepted that most plants containing a large number of polyphenols exhibit antioxidant activity [64–66]. Therefore, we checked our moss extracts toward their antioxidant potential. In order to determine total antioxidant capacity, ABTS and FRAP assays were used. Interestingly, among the samples tested, *D*. *pulvinatus* extract despite quite low TPC expressed the highest antioxidant potential. Moreover, comparison of the results obtained for mosses with these demonstrated by accepted medical plants possessing very high antioxidant potential indicated that antioxidant activity of mosses was possible to be considered high. However, antioxidant activity was not always correlated with the highest phenolics content (Table 6).

**Table 6:** TPC content and antioxidant activity of mosses extracts in comparison to accepted medical plants.

Species	Total phenolics mg GAE per g of FW	Antioxidant Activity	
		ABTS	FRAP
		Trolox equivalent [μM]	FeSO_4_equivalent [μM]
*Ceratodon purpureus*	1.71 ± 0.03	20.98 ± 0.02	771.33 ± 16.8
*Dryptodon pulvinatus*	0.70 ± 0.06	12.40 ± 0.62	549.4 ± 14.6
*Hypnum cupressiforme*	1.00 ± 0.02	7.59 ± 0.63	476.67 ± 6.62
*Rhytidiadelphus squarrosus*	0.65 ± 0.01	6.4 ± 0.72	412 ± 7.73
*Tortulla muralis*	1.04 ± 0.03	7.99 ± 0.14	469 ± 16.1
*Thymus vulgaris*	4.80 ± 0.26	13.10 ± 0.19	1092 ± 37
*Rosmarinus officinalis*	5.45 ± 0.06	13.62 ± 0.39	2076 ± 11
*Salvia officinalis*	2.23 ± 0.16	14.75 ± 0.11	1005 ± 10
*Mentha piperita*	1.38 ± 0.15	9.22 ± 0.69	788 ± 15

From group of phenolics the most numerous are flavonoids which play multiple functions in photoprotection and oxidative stress regulation, as well as in plant defense against herbivorous insects, fungi and other pathogens [67]. Flavonoids occur in many various compartments in mesophyll cells, including the nucleus, the chloroplasts and vacuoles, as well as in the epidermis [68]. It is wealthy to note that high light irradiance up-regulates the biosynthesis of dihydroxy B-ring-substituted flavonoids (such as luteolin-7-*O*-glycosides and quercetin-3-*O*-glycosides) whereas does not affect the biosynthesis of monohydroxy B-ring-substituted flavonoids (such as apigenin-7-*O*-glycosides and kaempferol-3-*O*-glycosides). Monohydroxy flavonoids, which have very similar UV-spectral features of their dihydroxy counterparts, predominate in plants growing under deep or partial shading [69]. Light-responsive dihydroxy flavonoids have much greater ability than their monohydroxy counterparts to inhibit the generation of ROS, and then quench ROS once they are formed. Thus the ratios of the ‘effective antioxidants’ to the ‘poor antioxidants’ significantly increase upon high light irradiance, irrespective of the relative proportions of different solar wavelengths reaching the leaf surface [70, 71]. Besides, the biosynthesis of effective antioxidant flavonoids is enhanced when plants growing under strong light are concomitantly faced with other stress agents [72, 73]. Lillo et al. [72] demonstrated that the content of flavonoids increases also in response to nitrogen and phosphorus depletion in plants. Obtained results confirmed these data because the highest antioxidant power was reported for species exposed to strong sunlight–*D*. *pulvinatus*.

Flavonoids as well as other phenolics have been reported as responsible also for antimicrobial effect of these extracts [36, 37, 61]. Observed activity is usually species-, extract type- and method-dependent, thus presented results are extremely different. Ertürk et al. [35] tested chosen mosses: *Ctenidium molluscum*, *Eurhynchium striatulum*, *Homalothecium lutescens*¸ *H*. *sericeum*, *Hypnum cupressiforme*, *Leucodon sciuroides*, *Thiudium delicatulum*, *Tomentypnum nitens* against 13 different microorganisms: four Gram-positive bacteria (*Bacillus cereus*, *Listeria monocytogenes*, *Clostridium perfringens*, and *Staphylococcus aureus*), six Gram-negative bacteria (*Escherichia coli*, *Salmonella* Typhimurium, *Klebsiella pneumoniae*, *Shigella sonnei*, *Yersinia enterocolitica*, and *Pseudomonas aeruginosa*) and three yeast strains (*Aspergillus niger*, *Candida albicans*, *Saccharomyces cerevisiae*) and showed that mosses may be used as possible natural antioxidant and antimicrobial agents to control various human, animal and plant disease. In this study two different methods: disc diffusion method and broth microdilution method were used to assess antimicrobial activity of moss samples collected from different locations of Turkey, including being also a subject of our study. Based on disc diffusion method and observed zones of microbial growth inhibition, antibacterial and antifungal activity of *H*. *cupressiforme* extract were stated. However, it is necessary to notice that the extract was used at a concentration of 20 mg/mL and its MIC against *S*. *aureus*, *E*. *coli* and *P*. *aeruginosa* tested by microdilution method reached above 25 mg/mL [35]. Thus, it’s no wonder that in our study the direct biostatic/biocidal activity of that and other extracts has not been demonstrated, when the highest concentration tested was 1.0 mg/mL. Furthermore, based on the results and well known limited bioavailability of plant-origin preparations in the tissues we proposed a completely different conclusion, that moss extracts tested did not possess antimicrobial activity at all. The more that our cytotoxic tests on mouse fibroblasts line L929 demonstrated cytotoxicity of the *T*. *muralis*, *H*. *cupressiforme* and *C*. *purpureus* extracts at a concentration above 0.5 mg/mL and the extracts from *D*. *pulvinatus* and *R*. *squarrosus* above 0.125 mg/mL, so their higher concentrations could not even be used *in vivo*. On the other hand, Manoj and Murugan [74] demonstrated, that methanol extract of *Plagiochila beddomei* Steph.–a liverwort expressed antimicrobial activity against a wide group of bacteria and fungi at much more lower concentrations. Its MICs reached since 0.0625 to 1 mg/mL, including MIC = 0.5 mg/mL against *S*. *aureus* and *E*. *coli*, MIC = 1 mg/mL against *P*. *aeruginosa* or MIC = 0.75 mg/mL against *C*. *albicans*. Aqua-acetonic and aqua-ethanolic extracts from other liverworts *Reboulia hemisphaerica* and *Marchantia palmata*, and from moss *Hydrogonium gracilantum* also possessed antibacterial activity at very low concentrations with MIC/MBC ranging from 0.00194 to 1 mg/mL [37]. In our study the extract from *D*. *pulvinatus* was an only one with similar activity, but just against *C*. *glabrata*. Probably it results from their different composition and a lower concentration of active components. Perhaps other method of extraction and the final product purification or enrichment could improve antimicrobial effect of our moss extracts.

*In vitro* cytotoxicity assays allows to evaluate the potential toxic effect of newly identified/obtained compounds or nature-derived products, which can affect basic functions of cells and through the interference of certain biological processes lead to dysfunctions and even death of cell. Continuous cell lines, as a simplified system, is a useful tool, which allows to minimize the influence of confounding factors. The choice of available types of cell lines for cytotoxicity tests is very large and rich [75–80]. One of the most commonly used cell lines in cytotoxicity testing is the fibroblast cell line L929 [81–85]. L929 fibroblasts are frequently used for testing multiple plant extract obtained for their potential to be profitable for a wide range of treatment [86, 87].

Literature directly relating to the cytotoxic properties of the moss extracts is rather poor, hence the direct comparison of the results obtained to the results of the work of other teams is quite difficult. Klavina et al. [5] noticed that from among several tested moss extracts these from *Sphagnum magellanicum*, *Dicranum polysetum* and *Pleurozium schreberi* showed the highest inhibitory activity (0.9–5 μg/mL), however it was depended on the kind of used cell line they were tested on. The rat glioma cells were markedly more sensitive to those extract than the other cell line: human lung carcinoma, mouse melanoma and human breast adenocarcinoma cell lines. Our extract tested on L929 cells showed generally lower cytotoxicity in comparison to those indicated above because even at 125 μg/mL the cells were mostly still alive. Interesting observation was made by Yağlıoğlu et al. [88]. They tested several extracts (a.o. hexane, CHCl_3_, EtOAc, MeOH) prepared from two species of mosses: the acrocarpous moss *Tortella tortuosa* (Hedw.) Limpr. and the pleurocarpous moss *Rhytidiadelphus triquetrus* (Hedw.) Warnst. and tested them using human cervix carcinoma (HeLa) and rat brain tumor (C6) cells. It has been found that the antiproliferative effect depended on the type of solvent, which was used to prepared the extracts. In the case of hexane and ethyl acetate extracts the level of proliferation inhibition was the highest (70–100%) starting already from 20 μg/mL up to 100 μg/mL (*R*. *triquetrus*), and in comparison to our extracts they were several time more cytotoxic. In contrast to that, the extracts of *T*. *tortuosa* caused only app. 20% decrease of cell proliferation even at the highest concentration thus they appear to be far less cytotoxic than our extracts, especially from this one prepared from *R*. *squarrosus*.

Phenolic compounds, widely distributed secondary metabolites in plants, form a group of molecules with highly diversified chemical structures. These phytocompounds are classified according to their carbon skeleton into the following main classes: simple phenols, phenolic acids, stilbenes, flavonoids, tannins [89]. The antioxidant activity of phenolic compounds depends on their structure. Generally, the antioxidant capacity is related to the number and the position of hydroxyl groups in the phenolic compound [62]. Moreover, the glycosylation or replacing of the hydroxyl group, or replacing this group by a methoxy group has a strongly suppressive influence on the antioxidant activity. Therefore, phenolic composition may have significant importance for the observed biological activity of plants. In the present study, a wide range of phenolic compounds, including hydroxycinnamic acids and different flavonoids sub-groups, were determined in two tested species of mosses such as *D*. *pulvinatus* and *R*. *squarrosus*. Both these mosses showed the presence of kaempferol-3-rutinoside, quercetin-3-rutinoside, and apigenin hexoside. Coumaroylquinic acid, sinapoyl hexoside, kaempferol-3-galactoside, procyanidin dimer and two eriodycitol hexoside were identified only in *D*. *pulvinatus*. In comparison, (+)-catechin, caffeic acid, 3,5-dicaffeoylquinic acid, and unknown kaempferol derivative were found in *R*. *squarrosus*. The presence of flavones in mosses is confirmed by other authors [5, 26, 88]. Phenolic acids such as coumaric, ferulic, gallic, caffeic, protocatechuic, cinnamic, sinapic, salicylic, chlorogenic and hydroxybenzoic acid were also found [74, 88]. Furthermore, the mosses can contain a series of polyhydroxylated coumarins [5, 25] as well as biflavonoids and hydroxybenzoic acid derivatives which havenot been studied in the present work. In addition, Klavina et al. [5] postulated that due to the high complexity of the chemical composition of mosses, there are no single substances that are specific for moss species or genus and are not influenced by environmental impacts.

Not only the phenolic compounds profiles but also the quantities of the identified phenolics were evidently different. Among identified compounds, kaempferol derivatives and sinapoyl hexoside were the dominant compounds in *D*. *pulvinatus* while apigenin hexoside and chlorogenic acid in *R*. *squarrosus*. The differences in the quantitative composition of phenolic components has been also confirmed by Klavina et al. [5] study conducted for 13 different species of mosses. For example, *p*-hydroxyacetophenone was the dominant phenolic compounds in *Sphagnum fallax* and 7,8-dixydroxy-5-metoxycoumarin-7-β-sophoroside in *Rhytidiadelphus triquetrus*.

A lot of so called medicinal plants, such as *Aloe vera*, *Calendula officinalis*, *Centella asiatica*, *Echinacea purpurea*, *Ginkgo biloba*, *Hypericum perforatum*, *Matricaria chamomilla*, *Lavandula angustifolia* or *Thymus vulgaris* and their products were described as able to support wound healing in both *in vitro* and *in vivo* studies. It was demonstrated, that they increased proliferation, migration, differentiation and secretory activity of fibroblast and keratinocytes, which are key cells during wound closure. The promotion of inflammatory cell infiltration, angiogenesis, extracellular matrix deposition and epithelialization in the presence of plant-derived products was also shown [89–93]. Therefore, we could also expected such pro-regenerative activity of the moss extracts tested. As it was shown in scratch assay, which simulated simplified process of wound healing, the extracts from *T*. *muralis* and *D*. *pulvinatus* intensified fibroblasts proliferation and thus wound closure. Manoj and Murugan [94] on animal model of skin wound also demonstrated, that methanolic and aqueous extracts from *Plagiochila beddomei* supported formation of granulation tissue, collagen production and angiogenesis, and observed changes were better than that in control group receiving Madecassol as reference drug. However, it is worth noting that only selected moss extracts possess a supportive effect for wound healing, which probably depends on their unique phytochemical composition.

In conclusion, the use of crude moss extracts as antimicrobial components of for instance ointments, dressings or disinfectants, is pointless. However, their species-specific stimulatory effect on fibroblasts migration at very low concentrations as well as antioxidant properties, entitles to consider further study on their regenerative activity and possible application as the preparations supporting wound healing.

## Supporting information

S1 Appendix Description of the studied moss species. (DOCX)

S1 Raw data ABTS, FRAP, TPC. (XLSX)

S2 Raw data L929. (XLS)
