# Supplementary material for: Cytotoxicity, antimicrobial and antioxidant activities of mosses obtained from open habitats
Source: PLoS One. 2021 Sep 20;16(9):e0257479. doi: 10.1371/journal.pone.0257479 (PMC8452054; doi:10.1371/journal.pone.0257479)
Supplement: S1 Appendix — (DOCX) [file pone.0257479.s001.docx]

**Appendix 1**

*Tortula muralis* – plant very small, to 1 cm long. Leaves twisted or curved when dry, margin strongly recurved. Apex obtuse or rounded, sometimes emarginate (Fig. 1 A). Cells quadrate-hexagonal, incrassate, papilose, 8-16(-20) µm wide. Seta purple, capsule erect (Smith 2001). This species is described as a characteristic to the temperate part of the southern hemisphere, boreal taiga zone, subcontinental, suboceanic and tropical region. It is cosmopolitan species, distributed in various climatical zones of most continents. *T*. *muralis* is growing from subneutral to basic, and moderately wet-moderately dry to dry environment, which prefers illuminated habitats. This species most common is listed on brick and stone walls, concrete and other manmade substrata, occasionally on natural rock-outcrops, most frequently on limestone (Dierβen 2001).

*Dryptodon* *pulvinatus* – plants to 3 cm, leaves erect, lanceolate to ovate-lanceolate, narrowed into hair-point (Fig. 1 B), margin recurved. Cells of the central part of the leaf quadrate, to quadrate-rectangular, 7-10 µm. Basal cells rectangular, 2-4 times as long as wide (Smith 2001). The range of this species is from Australia, and tropical region, to boreal taiga zone. *D*. *pulvinatus* is described as a cosmopolitan, a subcontinetal, and suboceanic species. Growing from in moderately acidic and basic environment, in moderately illuminated to in full light, and very dry habitat. Species is tolerant to atmospheric pollution. This species most common is listed on calcareous rock and concreto walls in urban areas, only occasionaly on trees (Dierβen 2001).

*Hypnum cupressiforme* – plant slender to robust. Stem irregularly pinnately branched and procumbent. Stem leaves concave, narrowly lanceolate, imbricate, abruptly narrowed acuminate to filiform apex (Fig. 1 C). Margin plane or recurved below, sub-entire or denticulate near apex. Cells of the central part of the leaf 5-9 × (32-)48-96 µm length (Smith 2001). This species is described as a subcontinental, suboceanic, characteristic to the temperate part of the southern hemisphere, arctic areas north of the polar forest limit, tundra biome, and even tropical region. *H*. *cupressiforme* it is cosmopolitan species, distributed in various climatical zones of most continents. Growing from highly acidic to subneutral, moderately wet-moderately dry to dry environment, which prefers illuminated habitats. This species most common is listed on trunks and lowr branches of trees, on logs, stumps, rock, on walls, roofs, but less frequently on soil (Dierβen 2001).

*Ceratodon purpureus* – plants in open to dense tufts, turfs, or mats, green, dark green, brownish green, light green or yellow-green, usually darker proximally, often tinged reddish brown or purple. Stems (0.2-)1-3(-4) cm. Leaves crowded (Fig. 1 D), erect-patent to contorted or somewhat crisped, rarely straight when dry, lanceolate, ovate-lanceolate, or triangular-lanceolate, 0.35-2.8 mm, margins recurved to near apex or rarely plane, irregularly serrate to uneven or smooth distally, apices acute to short-acuminate or, rarely, obtuse; costa strong, sub-percurrent to excurrent, sometimes as a long, smooth awn, medial laminal cells (6.5-)8-12(-14) µm, cell walls even, usually of medium thickness, often somewhat thicker and rounded at the cell angles. Seta 1-3(-4) cm, various shades of red, orange, or yellow. Capsule oblong to long-cylindric, (1-)2-2.5(-3) mm, smooth to strongly sulcate when dry; free to united at their nodes, finely papillose to spinulose-papillose, dark red and bordered to completely pale and absent borders. Spores (10-)11-14(-17) µm (Smith 2001).

*Rhytidiadelphus squarrosus* – plant robust, erect or ascending at least at tips, irregularly and sometimes sparsely pinnately branches. Stem leaves not plicate, strongly squarose (Fig. 1 E) and at shoot tips crowded, from erect, +/- sheathing broadly ovate basal part or strongly squarose, narrowed but not abruptly so to long acuminate apex. Leaves cells linear-elliptical, smotth, 6-9 × 40-80(-96) µm (Smith 2001). This species is described as a characteristic to the temperate part of the southern hemisphere, subcontinental, suboceanic, arctic areas north of the polar forest limit, tundra biome, circumpolar, occurring in more or less in the whole holarctic region, and Macaronesia. *R*. *squarossu* prefer moderately acid to subneutral, moderately wet-moderately dry environment, prefers illuminated habitats. This species most common is listed on grassy streamsides and banks in open woodland (Dierβen 2001).
